# Real-world palbociclib dose modifications and clinical outcomes in patients with HR+/HER2− metastatic breast cancer: A Flatiron Health database analysis

**DOI:** 10.1016/j.breast.2025.104448

**Published:** 2025-03-17

**Authors:** Rachel M. Layman, Xianchen Liu, Benjamin Li, Lynn McRoy, Adam Brufsky

**Affiliations:** a The University of Texas MD Anderson Cancer Center, 1515 Holcombe Blvd, Houston, TX, 77030, USA; b Pfizer Inc, 66 Hudson Yards, New York, NY, 10001, USA; c Department of Medicine, Division of Hematology/Oncology, UPMC Hillman Cancer Center, University of Pittsburgh Medical Center, 5115 Centre Ave, Pittsburgh, PA, 15232, USA

**Keywords:** Advanced breast cancer, Clinical outcomes, Cyclin-dependent kinase 4/6 inhibitor, Dose modifications, HR+/HER2−, Metastatic breast cancer, Palbociclib

## Abstract

**Purpose:**

To examine the associations of palbociclib dose modifications with clinical outcomes of patients with HR+/HER2− metastatic breast cancer (MBC) treated with first-line (1L) palbociclib + aromatase inhibitor (AI) in routine practice.

**Methods:**

Using the Flatiron Health Analytic Database, we conducted a retrospective analysis of HR+/HER2− MBC patients who started 1L palbociclib + AI February 2015–March 2020. Kaplan−Meier analyses were used to estimate treatment duration, real-world progression-free survival (rwPFS), and overall survival (OS) by palbociclib dose adjustments (any change in palbociclib daily dose while on treatment) and dose reductions (starting dose <125 mg/day or dose reduced while on treatment). Cox proportional hazard regression models were performed to compute unadjusted/adjusted hazard ratios (HRs).

**Results:**

Of 1302 patients with documented starting dose, 524 (40.2 %) had palbociclib dose adjustments; 778 (59.8 %) had none. Median treatment duration was significantly longer in patients with dose adjustments versus those with none (27.4 vs 21.4 months; adjusted HR = 0.80 [95 % CI, 0.69–0.93]; *P* = 0.004). Patients with and without dose adjustments showed similar median rwPFS (20.5 vs 19.6 months; adjusted HR = 0.89 [95 % CI, 0.76–1.04]; *P* = 0.133). Median OS was significantly prolonged in patients with versus without dose adjustments (57.8 vs 51.4 months; adjusted HR = 0.73 [95 % CI, 0.59–0.89]; *P* = 0.002). Similar findings were observed in patients with and without dose reductions.

**Conclusions:**

In this real-world study, rwPFS in HR+/HER2− MBC patients was maintained irrespective of dose adjustments. However, dose adjustments were associated with extended treatment duration and OS.

**Clinical trial registration:**

NCT05361655 (ClinicalTrials.gov)

## Highlights

• In patients with HR+/HER2− MBC, palbociclib dose modifications were common.
• Median rwPFS was maintained despite palbociclib dose modifications.
• Median OS was prolonged in those with palbociclib dose modifications.
• Palbociclib dose optimization may improve OS potentially due to longer treatment.

## Abbreviations

1L: first-line
AI: aromatase inhibitor
CDK4/6: cyclin-dependent kinase 4/6
CI: confidence interval
DFI: disease-free interval
EHR: electronic health record
ER+: estrogen receptor-positive
HR: hazard ratio
HR+/HER2–: hormone receptor-positive/human epidermal growth factor receptor 2-negative
MBC: metastatic breast cancer
NE: not estimable
OS: overall survival
PFS: progression-free survival
P-REALITY X: Palbociclib REAl-world first-LIne comparaTive effectiveness studY eXtended (NCT05361655)
rwPFS: real-world progression-free survival
TTNT: time-to-next treatment

## 1 Introduction

Cyclin-dependent kinase 4/6 (CDK4/6) inhibitor dose modification is recommended based on individual safety and tolerability during advanced and metastatic breast cancer (MBC) treatment [[1], [2], [3]]. In randomized clinical trials [[4], [5], [6], [7], [8], [9], [10]] and real-world studies [[11], [12], [13], [14], [15], [16], [17], [18], [19], [20], [21]] of CDK4/6 inhibitors in patients with hormone receptor-positive/human epidermal growth factor receptor 2-negative (HR+/HER2−) MBC, dose modifications have been found to be common. In the PALOMA-2 trial of palbociclib plus letrozole treatment in patients with estrogen receptor-positive (ER+)/HER2− MBC, dose reductions (39.4 %) in the first-line (1L) setting did not appear to compromise treatment efficacy, with no significant impact on progression-free survival (PFS) [8,10]. Some real-world studies have also reported that palbociclib dose reductions (occurring in approximately 20 %–60 % of study populations) did not significantly impact real-world PFS (rwPFS) [13,15,18,19]. However, a few studies have found palbociclib dose reductions to be associated with longer rwPFS or overall survival (OS), but in some instances the impact was no longer significant when adjusted for various patient characteristics or treatment patterns [15,20,22]. A more comprehensive analysis of palbociclib dose modifications in a larger patient population with longer follow-up is needed to provide a better understanding of the impact of dose modifications on patient outcomes.

P-REALITY X, the Palbociclib REAl-world first-LIne comparaTive effectiveness studY eXtended (NCT05361655), was a large analysis of a heterogeneous patient population in the United States with HR+/HER2− MBC from the Flatiron Health Analytics Database [23]. Patients had been treated in routine clinical practice with 1L palbociclib + aromatase inhibitor (AI) (n = 1324) or an AI alone (n = 1564) and followed for up to 68 months. The primary outcome of the P-REALITY X study, median OS, was significantly longer in patients treated with palbociclib + AI than in those treated with an AI alone (53.4 vs 40.4 months; hazard ratio [HR] = 0.67; *P* < 0.001); the secondary outcome, median rwPFS, was also significantly longer in those treated with palbociclib + AI (19.8 vs 13.9 months; HR = 0.68; *P* < 0.001).

Treatment patterns in P-REALITY X revealed that of the 1324 patients who were treated with palbociclib + AI, 83.8 % were started on palbociclib at 125 mg/day, 14.5 % initiated palbociclib at a lower dose, and 1.7% did not have documented dose information [24]. Herein, we report on the associations of these palbociclib dose adjustments (primary analysis) and dose reductions (secondary analysis) with clinical outcomes, including treatment duration, rwPFS, and OS.

## 2 Methods

### 2.1 Study design and data source

P-REALITY X was a real-world, retrospective analysis of a patient population with electronic health records (EHRs) from the Flatiron Health Analytic Database. The Flatiron Health database is a US nationwide dataset containing longitudinal deidentified patient data sourced from structured and unstructured EHRs. Detailed methods of P-REALITY X have been previously published [23].

For this analysis, data from the cohort of postmenopausal women and men who were treated with 1L palbociclib + AI in P-REALITY X were used. Key inclusion criteria were age ≥18 years, confirmed HR+/HER2− MBC, and a first prescription date (index date) for palbociclib + AI as 1L treatment of MBC between February 3, 2015 and March 31, 2020 (index period). Patients were followed from the start of index treatment until September 30, 2020 (data cutoff), death, or last medical activity, whichever came earliest.

### 2.2 Dose modifications: Adjustments and reductions

Per the prescribing information of palbociclib, the recommended starting dose is 125 mg/day [2]. For the primary analysis, a dose adjustment was defined as any change (increase or decrease) in the daily dose of palbociclib from any starting dose or previous dose while on treatment. This definition does not include patients who started palbociclib at a dose <125 mg/day and maintained that dose throughout the course of treatment. For the secondary analysis, a dose reduction was defined as a starting palbociclib dose <125 mg/day, regardless of whether the dose was changed later, or a dose reduction from the starting dose of 125 mg/day while on treatment. A supplementary analysis was also performed for patients with a starting palbociclib dose of <125 mg/day.

### 2.3 Study outcomes

The outcomes evaluated included treatment duration, rwPFS and OS. Treatment duration was defined as the number of months from the start to the end of palbociclib treatment. rwPFS was defined as the number of months from the start of palbociclib + AI to the first documentation of disease progression, evaluated based on clinical assessment or radiographic scan/tissue biopsy, last medical activity or death, whichever occurred first. OS was defined as the number of months from the start of treatment with palbociclib + AI until the date of death as previously described [23].

### 2.4 Statistical analyses

Patient demographic and disease characteristics at baseline were summarized by dose adjustments and reductions. Kaplan–Meier analyses were used to estimate median palbociclib treatment duration, rwPFS, and OS with 95 % confidence intervals (CIs). Unadjusted and adjusted HRs of palbociclib treatment duration, rwPFS, and OS were computed via univariable and multivariable Cox proportional hazard regression models. In the multivariable analyses, the baseline covariates adjusted for included age, sex, race/ethnicity, healthcare practice type, disease stage at initial diagnosis, Eastern Cooperative Oncology Group performance status, visceral metastases, bone-only metastases, number of metastatic sites and disease-free interval (DFI) from initial breast cancer to MBC diagnosis. A sensitivity analysis was conducted by comparing restricted mean survival times in palbociclib treatment duration, rwPFS, and OS between the respective cohorts [25].

## 3 Results

### 3.1 Patient characteristics

During the index period of P-REALITY X, a total of 1324 patients were treated with palbociclib + AI as 1L therapy; mean age was 67 years, 99.2 % were women, and 68.0 % were White [23]. Of the 1302 (98.3 %) patients who had their starting palbociclib dose documented, 524 (40.2 %) had palbociclib dose adjustments, while 778 (59.8 %) had no dose adjustments. The median time from palbociclib start to a first dose adjustment was 85 days; 25 % of patients experienced a first dose adjustment at a median time of 52 days and 75 % experienced dose adjustments at a median time of 196 days.

As shown in Table 1, mean ages of patients with and without dose adjustments were similar, at 67.4 and 67.0 years, respectively. Patient demographic and disease characteristics were generally similar across these patient groups, except that the dose adjustment group compared with the no dose adjustment group had more White patients (71.8 % vs 65.8 %), more patients with multiple health plans (32.6 % vs 26.7 %), and more patients with a DFI from initial breast cancer to MBC diagnosis of >5 years (44.7 % vs 39.6 %). The median follow-up duration was longer in patients with dose adjustments than in those without (28.5 vs 22.6 months).

**Table 1:** Demographic and disease characteristics: patients with palbociclib dose adjustments^a^ versus patients with no dose adjustments.

Characteristics	Dose Adjustments n = 524	No Dose Adjustments n = 778
**Age, years**		
Mean (SD)	67.4 (9.2)	67.0 (9.7)
Median (IQR)	68 (61–74)	67 (60–74)
**Age group, n (%)**		
18–49 years	16 (3.1)	28 (3.6)
50–64 years	186 (35.5)	276 (35.5)
65–74 years	201 (38.4)	289 (37.1)
≥ 75 years	121 (23.1)	185 (23.8)
**Sex, n (%)**		
Female	520 (99.2)	772 (99.2)
Male	4 (0.8)	6 (0.8)
**Race/ethnicity, n (%)**		
White	376 (71.8)	512 (65.8)
Black	30 (5.7)	75 (9.6)
Other/unknown^b^	118 (22.5)	191 (24.6)
**Healthcare practice type, n (%)**		
Community	475 (90.6)	715 (91.9)
Academic	49 (9.4)	63 (8.1)
**Health plan, n (%)**		
Commercial health plan + any other	171 (32.6)	208 (26.7)
Commercial health plan	125 (23.9)	197 (25.3)
Medicare	21 (4.0)	38 (4.9)
Medicaid	5 (1.0)	11 (1.4)
Other payer type	202 (38.5)	324 (41.6)
**Disease stage at initial diagnosis, n (%)**		
I	66 (12.6)	79 (10.2)
II	138 (26.3)	200 (25.7)
III	62 (11.8)	114 (14.7)
IV	213 (40.6)	321 (41.3)
Not documented	45 (8.6)	64 (8.2)
**ECOG performance status, n (%)**		
0	207 (39.5)	284 (36.5)
1	118 (22.5)	195 (25.1)
2, 3, or 4	56 (10.7)	96 (12.3)
Not documented	143 (27.3)	203 (26.1)
**Visceral metastases,**^c^**n (%)**	186 (35.5)	252 (32.4)
**Bone-only metastases,**^d^**n (%)**	213 (40.6)	299 (38.4)
**Brain metastases, n (%)**	12 (2.3)	14 (1.8)
**Number of metastatic sites,**^e^**n (%)**		
1	253 (48.3)	389 (50.0)
2	137 (26.1)	225 (28.9)
3	80 (15.3)	95 (12.2)
4	26 (5.0)	30 (3.9)
≥ 5	15 (2.9)	18 (2.3)
Not documented	13 (2.5)	21 (2.7)
**Number of metastatic sites in patients with ≥ 1 metastatic site**		
Mean (SD)	1.9 (1.1)	1.8 (1.0)
Median (IQR)	2.0 (1.0–2.0)	1.0 (1.0–2.0)
Missing	13	21
**DFI from initial breast cancer to MBC diagnosis, n (%)**		
De novo	213 (40.6)	321 (41.3)
≤ 1 year	10 (1.9)	29 (3.7)
> 1−5 years	66 (12.6)	120 (15.4)
> 5 years	234 (44.7)	308 (39.6)
Not documented	1 (0.2)	0
**NCI comorbidity index**		
Mean (SD)	0.3 (0.5)	0.3 (0.5)
Median (IQR)	0.0 (0.0–0.5)	0.0 (0.0–0.5)
**First-line AI, n (%)**		
Letrozole	458 (87.4)	663 (85.2)
Anastrozole	55 (10.5)	87 (11.2)
Exemestane	11 (2.1)	28 (3.6)

Abbreviations: AI, aromatase inhibitor; DFI, disease-free interval; ECOG, Eastern Cooperative Oncology Group; IQR, interquartile range; MBC, metastatic breast cancer; NCI, National Cancer Institute; SD, standard deviation.

a A dose adjustment was defined as any change (increase or decrease) in the daily dose of palbociclib from any starting dose or previous dose while on treatment.

b Other/unknown also includes Asian, Hispanic or Latino.

c Visceral disease was defined as metastatic disease in the lung and/or liver; patients could have other sites of metastases. No visceral disease was defined as no lung or liver metastases.

d Bone-only disease was defined as metastatic disease in the bone only.

e Multiple metastases at the same site were counted as one site (e.g., if a patient had three bone metastases in the spine, it was considered only one site).

Of the 1302 patients who had their starting palbociclib dose documented, 192 (14.7 %) started at < 125 mg/day (n = 144 at 100 mg/day; n = 48 at 75 mg/day), while 1110 (85.3 %) started at 125 mg/day. Of the 144 patients who started at 100 mg/day, 53 (36.8 %) experienced dose adjustments and of the 48 patients who started at 75 mg/day, 15 (31.3 %) experienced dose adjustments. Among those who started at 125 mg/day, 456 (41.1 %) had at least one dose reduction. The palbociclib dose reduction group (n = 648) included patients who started palbociclib at < 125 mg/day (n = 192) and those who started at 125 mg/day and who had at least one dose reduction (n = 456). A total of 654 (50.2 %) patients started and maintained palbociclib at 125 mg/day (i.e., no dose reductions). Patients who had their palbociclib dose reduced were older compared with those who maintained palbociclib at 125 mg/day (68.2 vs 66.1 years; 26.7 % vs 20.3 % were ≥75 years of age), and were more likely to have multiple health plans (31.8 % vs 26.5 %), bone-only metastases (41.0 % vs 37.6 %), and DFI from initial breast cancer to MBC diagnosis of >5 years (43.7 % vs 39.6 %; Table 2). Other patient demographic and disease characteristics were generally similar. The median follow-up duration was longer in patients with a reduced dose than in those without dose reductions (26.9 vs 22.6 months).

**Table 2:** Demographic and disease characteristics: patients with palbociclib dose reductions^a^ versus patients with maintained 125 mg/day dose.

Characteristics	<125 mg/day n = 648	125 mg/day n = 654
**Age, years**		
Mean (SD)	68.2 (9.3)	66.1 (9.5)
Median (IQR)	69 (62–75)	66 (60–73)
**Age group, n (%)**		
18–49 years	17 (2.6)	27 (4.1)
50–64 years	210 (32.4)	252 (38.5)
65–74 years	248 (38.3)	242 (37.0)
≥ 75 years	173 (26.7)	133 (20.3)
**Sex, n (%)**		
Female	644 (99.4)	648 (99.1)
Male	4 (0.6)	6 (0.9)
**Race/ethnicity, n (%)**		
White	447 (69.0)	441 (67.4)
Black	39 (6.0)	66 (10.1)
Other/unknown^b^	162 (25.0)	147 (22.5)
**Healthcare practice type, n (%)**		
Community	594 (91.7)	596 (91.1)
Academic	54 (8.3)	58 (8.9)
**Health plan, n (%)**		
Commercial health plan + any other	206 (31.8)	173 (26.5)
Commercial health plan	161 (24.8)	161 (24.6)
Medicare	30 (4.6)	29 (4.4)
Medicaid	5 (0.8)	11 (1.7)
Other payer type	246 (38.0)	280 (42.8)
**Disease stage at initial diagnosis, n (%)**		
I	75 (11.6)	70 (10.7)
II	166 (25.6)	172 (26.3)
III	79 (12.2)	97 (14.8)
IV	268 (41.4)	266 (40.7)
Not documented	60 (9.3)	49 (7.5)
**ECOG performance status, n (%)**		
0	242 (37.3)	249 (38.1)
1	153 (23.6)	160 (24.5)
2, 3, or 4	79 (12.2)	73 (11.2)
Not documented	174 (26.9)	172 (26.3)
**Visceral metastases,**^c^**n (%)**	222 (34.3)	216 (33.0)
**Bone-only metastases,**^d^**n (%)**	266 (41.0)	246 (37.6)
**Brain metastases, n (%)**	13 (2.0)	13 (2.0)
**Number of metastatic sites,**^e^**n (%)**		
1	318 (49.1)	324 (49.5)
2	169 (26.1)	193 (29.5)
3	98 (15.1)	77 (11.8)
4	28 (4.3)	28 (4.3)
≥ 5	18 (2.8)	15 (2.3)
Not documented	17 (2.6)	17 (2.6)
**Number of metastatic sites in patients with ≥ 1 metastatic site**		
Mean (SD)	1.8 (1.1)	1.8 (1.0)
Median (IQR)	1.0 (1.0–2.0)	1.0 (1.0–2.0)
Missing	17	17
**DFI from initial breast cancer to MBC diagnosis, n (%)**		
De novo	268 (41.4)	266 (40.7)
≤ 1 year	14 (2.2)	25 (3.8)
> 1–5 years	82 (12.7)	104 (15.9)
> 5 years	283 (43.7)	259 (39.6)
Not documented	1 (0.2)	0
**NCI comorbidity index**		
Mean (SD)	0.3 (0.5)	0.3 (0.5)
Median (IQR)	0.0 (0.0–0.5)	0.0 (0.0–0.5)
**First-line AI, n (%)**		
Letrozole	557 (86.0)	564 (86.2)
Anastrozole	75 (11.6)	67 (10.2)
Exemestane	16 (2.5)	23 (3.5)

Abbreviations: AI, aromatase inhibitor; DFI, disease-free interval; ECOG, Eastern Cooperative Oncology Group; IQR, interquartile range; MBC, metastatic breast cancer; NCI, National Cancer Institute; SD, standard deviation.

a A dose reduction was defined as a starting palbociclib dose <125 mg/day, regardless of whether the dose was changed later, or a dose reduction from the starting dose of 125 mg/day while on treatment.

b Other/unknown also includes Asian, Hispanic or Latino.

c Visceral disease was defined as metastatic disease in the lung and/or liver; patients could have other sites of metastases. No visceral disease was defined as no lung or liver metastases.

d Bone-only disease was defined as metastatic disease in the bone only.

e Multiple metastases at the same site were counted as one site (e.g., if a patient had three bone metastases in the spine, it was considered only one site).

### 3.2 Outcomes by dose adjustments

**Fig. 1:**
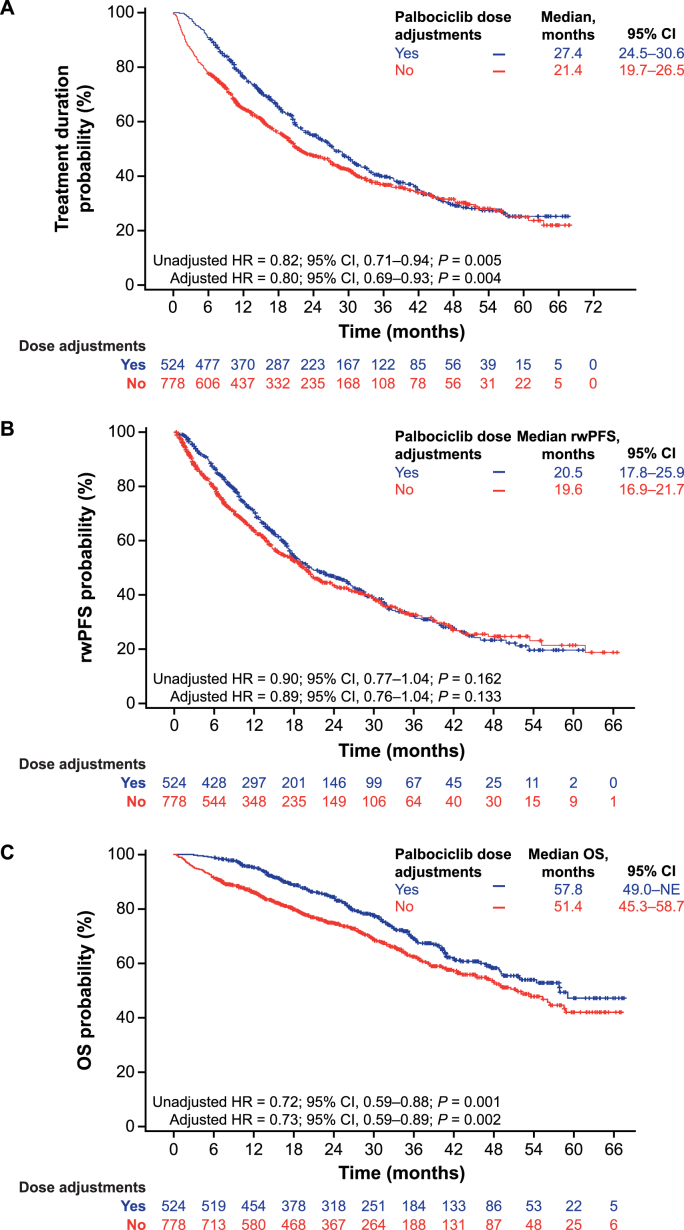
Kaplan–Meier curves of palbociclib treatment duration (A), rwPFS (B) and OS (C) by palbociclib dose adjustments. Abbreviations: CI, confidence interval; HR, hazard ratio; NE, not estimable; OS, overall survival; rwPFS, real-world progression-free survival.

Median palbociclib treatment duration was longer in patients with dose adjustments than in those with no dose adjustments (27.4 vs 21.4 months; unadjusted HR = 0.82 [95 % CI, 0.71–0.94]; *P* = 0.005; adjusted HR = 0.80 [95 % CI, 0.69–0.93]; *P* = 0.004; Fig. 1A). Patients with and without dose adjustments had similar median rwPFS (20.5 vs 19.6 months; unadjusted HR = 0.90 [95 % CI, 0.77–1.04]; *P* = 0.162; adjusted HR = 0.89 [95 % CI, 0.76–1.04]; *P* = 0.133; Fig. 1B). Median OS was significantly prolonged in patients with dose adjustments compared with those with no dose adjustments (57.8 vs 51.4 months), with an estimated 27 % reduction in the risk of death after multivariable analysis (unadjusted HR = 0.72 [95 % CI, 0.59–0.88]; *P* = 0.001; adjusted HR = 0.73 [95 % CI, 0.59–0.89]; *P* = 0.002; Fig. 1C).

The restricted mean survival times between patients with and without dose adjustments were 33.7 versus 30.4 months (*P* = 0.040) for palbociclib treatment duration, 27.9 versus 27.0 months (*P* = 0.540) for rwPFS, and 49.0 versus 44.5 months (*P* = 0.003) for OS.

### 3.3 Outcomes by dose reductions

**Fig. 2:**
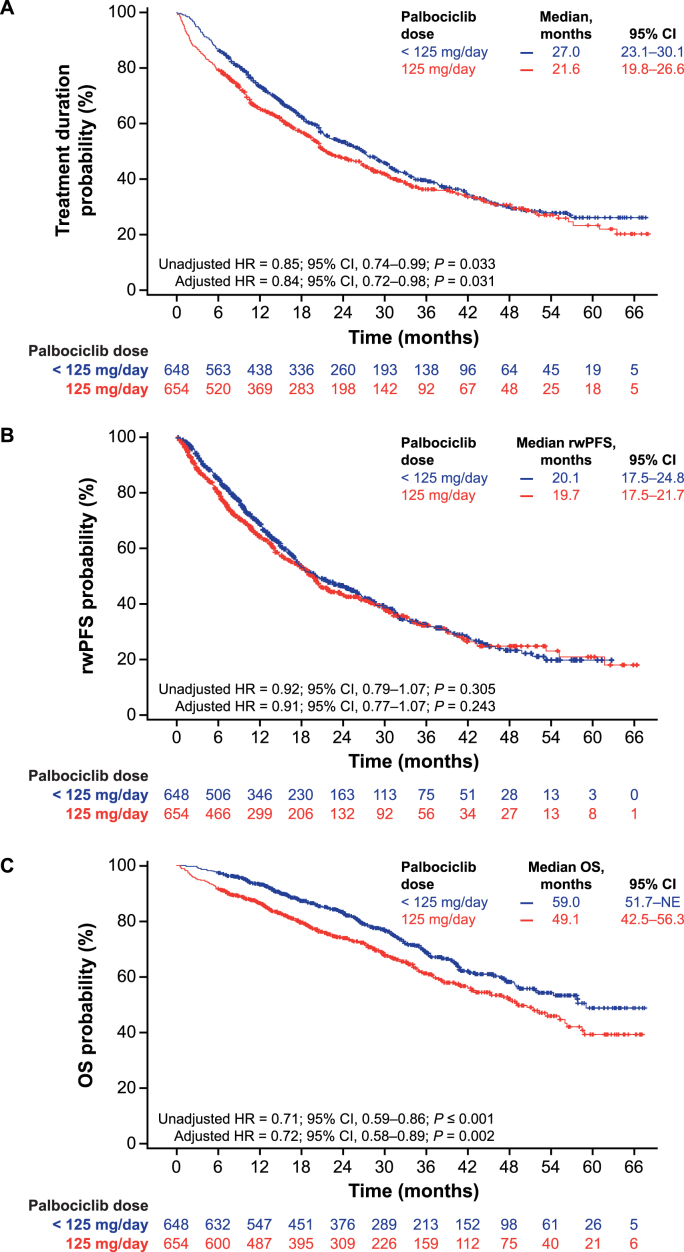
Kaplan–Meier curves of palbociclib treatment duration (A), rwPFS (B) and OS (C) by palbociclib dose. Abbreviations: CI, confidence interval; HR, hazard ratio; NE, not estimable; OS, overall survival; rwPFS, real-world progression-free survival.

Median treatment duration was longer in patients with palbociclib dose reductions compared with those who started and maintained a 125 mg/day dose (27.0 vs 21.6 months; unadjusted HR = 0.85 [95 % CI, 0.74–0.99]; *P* = 0.033; adjusted HR = 0.84 [95 % CI, 0.72–0.98]; *P* = 0.031; Fig. 2A). Patients with and without dose reductions of palbociclib had similar median rwPFS (20.1 vs 19.7 months; unadjusted HR = 0.92 [95 % CI, 0.79–1.07]; *P* = 0.305; adjusted HR = 0.91 [95 % CI, 0.77–1.07]; *P* = 0.243; Fig. 2B). Median OS was significantly prolonged in patients with palbociclib dose reductions compared with those who started and maintained a 125 mg/day dose (59.0 vs 49.1 months), with an estimated 28 % reduction in the risk of death after multivariable analysis; unadjusted HR = 0.71 [95 % CI, 0.59–0.86]; *P* < 0.001; adjusted HR = 0.72 [95 % CI, 0.58–0.89]; *P* = 0.002; Fig. 2C). For patients who started palbociclib at < 125 mg/day, median treatment duration was 25.0 months, median rwPFS was 17.8 months and median OS was not reached (Supplementary Fig. S1).

The restricted mean survival times between patients with and without dose reductions were 33.0 versus 30.3 months (*P* = 0.088) for palbociclib treatment duration, 27.9 versus 27.2 months (*P* = 0.656) for rwPFS, and 48.8 versus 43.8 months (*P* = 0.001) for OS.

## 4 Discussion

Similar to treatment of HR+/HER2− MBC with other CDK4/6 inhibitors in real-world settings [12,14,16,21], palbociclib dose adjustments were common in patients in the US Flatiron Health Analytic Database. Approximately 40 % of patients treated with palbociclib + AI experienced dose adjustments regardless of starting dose; 41 % experienced at least one dose reduction from their initial dose of 125 mg/day. The proportions of patients with palbociclib dose reductions from their initial dose of 125 mg/day were similar to that reported in the PALOMA-1 (40 %) [26] and PALOMA-2 (39 %) [10] trials, both of which evaluated palbociclib + letrozole versus placebo + letrozole in the 1L setting in postmenopausal women with ER+/HER2− MBC. These rates of palbociclib dose reductions are numerically lower than what was reported in the MONARCH-3 trial of abemaciclib (43 %) [27] and the MONALEESA-2 trial of ribociclib (58 %) [5] in the 1L treatment setting. In the current study, approximately 15 % of patients treated with palbociclib had a reduced starting dose (100/75 mg/day); per protocols, this did not occur in the clinical trial setting [2,10,26].

In this large real-world data analysis, we found that dose adjustments/reductions were not significantly associated with disease progression. In fact, across patient groups with and without dose adjustments/reductions, median rwPFS was approximately 20 months with variations of less than 1 month between study groups. This rwPFS is quite similar to that reported in P-REALITY X (19.8 months) in the overall population treated with palbociclib + AI [23], and our study findings are consistent with those from the PALOMA-2 trial [8], in which palbociclib dose reductions also did not signficantly impact PFS. However, we did find that dose modifications were associated with significantly longer median treatment duration (approximately 6 months) and prolonged median OS (ranged 6–10 months). These study findings provide further real-world evidence that dose modifications of palbociclib in the 1L setting do not compromise treatment effectiveness in patients with HR+/HER2− MBC and may be associated with an OS benefit potentially due to extended treatment duration.

Potential explanations for the longer treatment duration in patients with dose modifications may be due to dose optimization; patients may have had increased tolerability, experienced fewer adverse events, and have been less likely to have discontinued palbociclib early than patients without dose modifications [15]. A real-world, Italian multicenter study (n = 375) reported that patients receiving reduced palbociclib doses had a longer time-to-treatment discontinuation than those with no dose reductions (palbociclib + fulvestrant: 17.7 vs 9.2 months; palbociclib + AI: 16.6 vs 7.4 months) [28]. In addition, some patients may have experienced an adverse event that triggered a dose modification, which, to some extent, could have contributed to a longer treatment duration. For example, to manage Grade 3 neutropenia, palbociclib should be withheld until recovery to Grade ≤2 and then resumed at the next lower dose [2]. Although dose interruptions due to management of adverse events may have affected treatment duration in this study, they likely did not account for the several months longer treatment duration among those with dose modifications, since in PALOMA-2, the average number of dose interruptions was 2.0 per patient, lasting on average 4 days [10], and median duration of a Grade ≥3 neutropenia episode was 7.0 days [9].

Most findings from observational, real-world studies show consistent median rwPFS, irrespective of dose reductions, in patients with HR+/HER2− MBC treated with palbociclib [13,15,18,19]. However, one German, multicenter study of 448 patients treated with a CDK4/6 inhibitor (71 % with palbociclib) reported that in patients with a dose reduction (approximately 30 %), rwPFS was significantly longer than in those without dose reductions (24 vs 15 months) [22]. Explanations for these contrasting findings may include a younger population (mean age: 63 vs 67 years), an undetermined HR status (15 % of patients), use of a CDK4/6 inhibitor other than palbociclib (∼30 % of patients), treatment with a CDK4/6 inhibitor + fulvestrant (35 % of patients), and treatment in second-line or later lines (38 % of patients) [22]. In another real-world study by Ismail et al. [15], in which 33 % of 598 patients with HR+/HER2− MBC had their palbociclib dose reduced, the time-to-next treatment (TTNT), used as a surrogate for PFS, was also longer compared with those without dose reductions (16.9 vs 11.4 months). However, this longer TTNT may have been related to a longer palbociclib treatment duration. A longer rwPFS in patients with versus without dose reductions of palbociclib was also reported in a US study conducted at a single academic institution, but the difference was not significant after adjusting for patient characteristics [20].

In the current study, patients with dose modifications had a median OS that was approximately 6/10 months longer than in those without dose adjustments/reductions. This is likely related to the longer treatment duration seen in patients with dose modifications, which was approximately 6 months longer than in those without dose modifications. In the Ismail et al. study [15], they also found that median OS was prolonged in patients with dose reductions compared with patients without dose reductions (29.7 vs 21.9 months, *P* = 0.003). However, when they excluded patients with early discontinuation of palbociclib (23 % of patients), median OS was no longer significantly different (*P* = 0.75).

In recent years, there has been increasing recognition in oncology that dose optimization is needed, especially for therapies that patients may take for several months or years [29]. This greater awareness of patients' need for long-term tolerability and safety of oncology drugs motivated the US Food and Drug Administration's Oncology Center of Excellence to introduce Project Optimus in 2021, which aims to reform dose selection and optimization in oncology drug development [[30], [31], [32]]. Other initiatives by patient-advocate groups are focused on incorporating patient-reported outcomes into drug development and precision medicine to better understand oncologic drug safety and tolerability and the relationship to dosing optimization [30,33]. In a 2020 patient advocate-led survey of over 1200 patients with MBC, 86 % reported experiencing ≥1 significant treatment-related side effect; 46 % received a dose reduction to manage their side effect, with most (83 %) reporting improvement [30]. Notably, 92 % of patients responded that they were willing to discuss flexible dosing options with their providers so that their quality of life may be maintained or improved. Our findings in this study of patients with HR+/HER2− MBC treated with palbociclib + AI in routine clinical practice provide further evidence that dose modification does not compromise the effectiveness of palbociclib. Such findings may encourage providers of breast cancer care to speak with their patients on dose optimization according to their individual needs leading to improved shared decision-making and patients' well-being [30].

P-REALITY X was a large study of a diverse patient population with HR+/HER2− MBC with a long median follow-up across multiple sites in the real-world setting. As a retrospective observational study of EHRs, inherent limitations include missing, incomplete and erroneously entered patient data. Also, no causal relationship could be made between dose adjustments/reductions and the outcomes evaluated in the study. Furthermore, disease progression was assessed by treating physicians and may not have used standardized criteria. Although multivariable adjusted analyses of outcomes were conducted in this study, other unevaluated patient and disease characteristics may have affected clinical outcomes. Dose adjustments/reductions were defined based on prescriptions, and it cannot be confirmed how patients actually took the medication. Reasons for dose modifications of palbociclib, such as adverse events, were not captured in this study. Immortal time bias is a potential concern with retrospective database analyses of dose modifications and clinical outcomes. However, in our analyses of palbociclib treatment duration, rwPFS, and OS, immortal time bias would be less likely to occur or would not have been a major concern since the outcomes of treatment discontinuation and death occurred after dose modifications and very few patients discontinued treatment, had disease progression or died early on in the study period [15,[34], [35], [36], [37]]. Indeed, the median time to first dose adjustment was less than 3 months (median = 85 days), markedly shorter than median time to treatment discontinuation (>20 months), median rwPFS (∼20 months) and median OS (>50 months) (Fig. 1, Fig. 2). However, given this is a retrospective database analysis, prospective studies, especially randomized clinical trials, are warranted to determine whether there is a causal relationship between dose modifications and outcomes [34]. In Cox proportional hazard regressions, violation of the proportional hazard assumption may lead to biased findings. However, the results of our sensitivity analysis, which compared restricted mean survival times between patients with and without dose modifications, supported the conclusions based on Cox regression models. Lastly, although the sample size is large and the patient population represented in the Flatiron Health database is more diverse than that frequently included in clinical trials, results may not be generalized to other populations outside the Flatiron Health network.

## 5 Conclusions

In this large real-world study of ∼1300 patients with HR+/HER2− MBC, rwPFS was maintained irrespective of palbociclib dose adjustments that occurred in approximately 40 % of those treated with 1L palbociclib + AI. Furthermore, patients with dose modifications had extended treatment duration, as well as prolonged OS in the real-world setting. Such findings suggest palbociclib dose optimization may be associated with improved OS, potentially, in part due to longer treatment duration. Future research is warranted on the association of dose optimization with OS and the reasons behind palbociclib dose modifications to better understand patient populations in need of dose optimization.

## CRediT authorship contribution statement

**Rachel M. Layman:** Writing – review & editing, Writing – original draft, Conceptualization. **Xianchen Liu:** Writing – review & editing, Writing – original draft, Formal analysis, Data curation, Conceptualization. **Benjamin Li:** Writing – review & editing, Writing – original draft, Formal analysis, Data curation, Conceptualization. **Lynn McRoy:** Writing – review & editing, Writing – original draft, Conceptualization. **Adam Brufsky:** Writing – review & editing, Writing – original draft, Conceptualization.

## Data availability statement

Upon request, and subject to review, Pfizer will provide the data that support the findings of this study. Subject to certain criteria, conditions and exceptions, Pfizer may also provide access to the related individual de–identified participant data. See https://www.pfizer.com/science/clinical-trials/data-and-results https://www.pfizer.com/science/clinical-trials/trial-data-and-results for more information.

## Ethics statement

This retrospective database analysis was conducted in accordance with the Guidelines for Good Pharmacoepidemiology Practice, Good Practices for Outcomes Research issued by the International Society for Pharmacoeconomics and Outcomes Research, and Good Practices for Real-world Data Studies of Treatment and/or Comparative Effectiveness. As this study is retrospective and non-interventional and uses anonymized data, it is exempt from institutional review board approval and included a waiver of informed consent. Trial registration number: NCT05361655.

## Funding

This study was sponsored by Pfizer Inc.

## Declaration of competing interest

The authors declare the following financial interests/personal relationships which may be considered as potential competing interests:

Rachel M. Layman: reports advisory/consultancy fees from Novartis, Eli Lilly, Gilead Sciences, Biotheryx and Celcuity; honorarium provided by Pfizer Inc; institutional research support from Pfizer Inc, Novartis, Eli Lilly, Puma, Celcuity, Accutar Biotechnology and Arvinas.

Xianchen Liu: reports employee of, and stockholder in Pfizer Inc.

Benjamin Li: reports employee of, and stockholder in Pfizer Inc.

Lynn McRoy: reports employee of, and stockholder in Pfizer Inc.

Adam Brufsky: reports consultancy fees from Pfizer Inc, Novartis and Eli Lilly.
