# Supplementary material for: Real-world palbociclib dose modifications and clinical outcomes in patients with HR+/HER2− metastatic breast cancer: A Flatiron Health database analysis
Source: Breast. 2025 Mar 17;81:104448. doi: 10.1016/j.breast.2025.104448 (PMC11986547; doi:10.1016/j.breast.2025.104448)
Supplement: Multimedia component 1 [file mmc1.docx]

**Supplementary Material**

**Supplementary Fig. S1 Kaplan–Meier curves of palbociclib treatment duration (A), rwPFS (B) and OS (C) for patients with palbociclib starting dose < 125 mg/day (n = 192)**

**
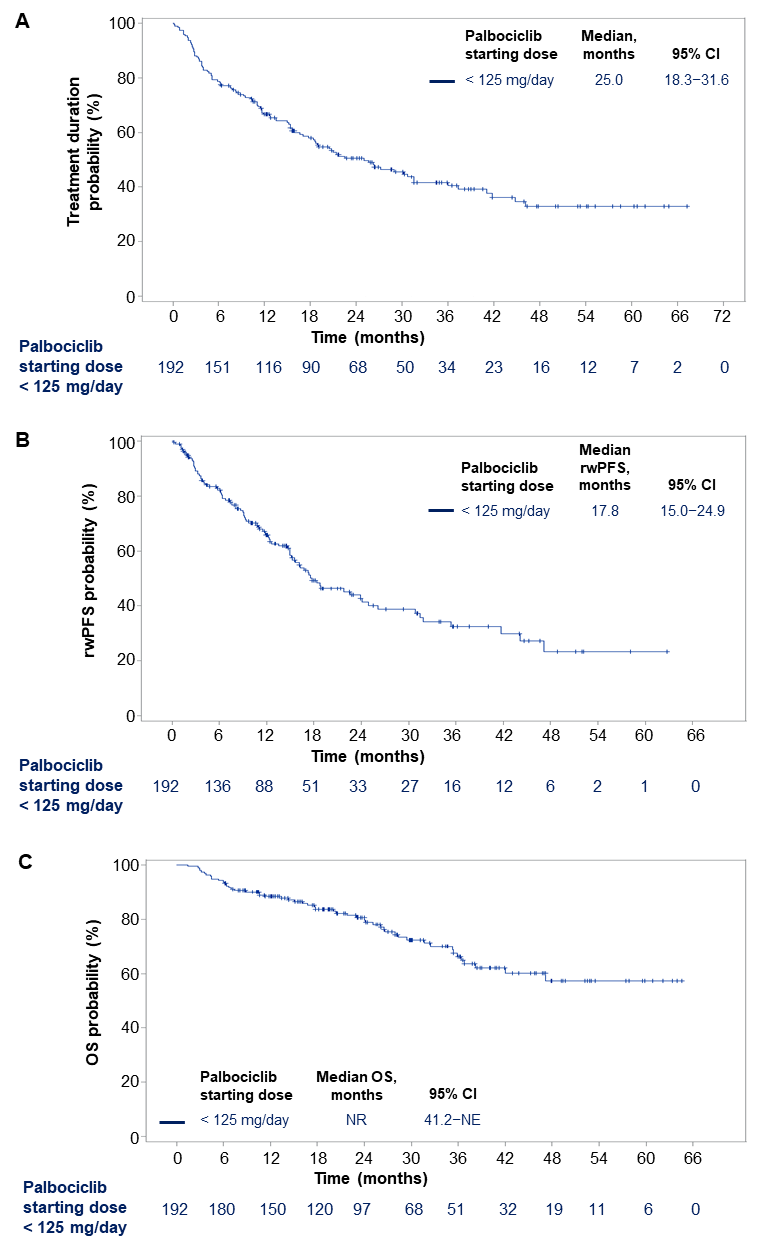
**

Abbreviations: CI, confidence interval; HR, hazard ratio; NE, not estimable; NR, not reached; OS, overall survival; rwPFS, real-world progression-free survival.
